# CRISPR/Cas9 and next generation sequencing in the personalized treatment of Cancer

**DOI:** 10.1186/s12943-022-01565-1

**Published:** 2022-03-24

**Authors:** Sushmaa Chandralekha Selvakumar, K. Auxzilia Preethi, Kehinde Ross, Deusdedit Tusubira, Mohd Wajid Ali Khan, Panagal Mani, Tentu Nageswara Rao, Durairaj Sekar

**Affiliations:** 1 Centre for Cellular and Molecular Research, Saveetha Dental College and Hospitals, Saveetha Institute of Medical and Technical Sciences (SIMATS), Saveetha University, Chennai, Tamil Nadu 600077 India; 2 School of Pharmacy and Biomolecular Sciences, Liverpool John Moores University, Liverpool, UK; 3 Biochemistry Department, Mbarara University of Science and Technology, Mbarara, Uganda; 4 Department of Chemistry, College of Sciences, University of Ha’il, Ha’il, 2440 Saudi Arabia; 5 Department of Biotechnology, Annai College of Arts and Science, Kumbakonam, Tamilnadu, India; 6 Department of Chemistry, Krishna University, Machilipatnam, Andhra Pradesh 521001 India

**Keywords:** CRISPR/Cas9, Next generation sequencing (NGS), Personalized medicine, Liquid biopsy, Genome editing

## Abstract

**Background:**

Cancer is caused by a combination of genetic and epigenetic abnormalities. Current cancer therapies are limited due to the complexity of their mechanism, underlining the need for alternative therapeutic approaches. Interestingly, combining the Clustered Regularly Interspaced Short Palindromic Repeats (CRISPR/Cas9) system with next-generation sequencing (NGS) has the potential to speed up the identification, validation, and targeting of high-value targets.

**Main text:**

Personalized or precision medicine combines genetic information with phenotypic and environmental characteristics to produce healthcare tailored to the individual and eliminates the constraints of “one-size-fits-all” therapy. Precision medicine is now possible thanks to cancer genome sequencing. Having advantages over limited sample requirements and the recent development of biomarkers have made the use of NGS a major leap in personalized medicine. Tumor and cell-free DNA profiling using NGS, proteome and RNA analyses, and a better understanding of immunological systems, are all helping to improve cancer treatment choices. Finally, direct targeting of tumor genes in cancer cells with CRISPR/Cas9 may be achievable, allowing for eliminating genetic changes that lead to tumor growth and metastatic capability.

**Conclusion:**

With NGS and CRISPR/Cas9, the goal is no longer to match the treatment for the diagnosed tumor but rather to build a treatment method that fits the tumor exactly. Hence, in this review, we have discussed the potential role of CRISPR/Cas9 and NGS in advancing personalized medicine.

## Introduction

Cancer is one of the world’s foremost causes of morbidity and mortality where several signaling pathways, linked to cell proliferation, angiogenesis, metastasis, and resistance to apoptosis evasion are dysregulated [1]. Current cancer therapies like chemotherapy, radiation therapy, and surgery are limited to certain patients. This is because not all tumors are caused by the same mutations in the genome and the tumor varies for each individual. Due to the complexity of their mechanism, the need for alternative therapeutic approaches is gaining attention. Interestingly, genome editing has emerged as a therapeutic tool for various diseases [2]. In particular, Clustered Regularly Interspaced Short Palindromic Repeats (CRISPR/Cas9) has been studied for the treatment of Non-Small Cell Lung Carcinoma (NSCLC), breast cancer, multiple myeloma, glioblastoma, leukemia, and so on. Combining the CRISPR/Cas9 system with next-generation sequencing (NGS) has the potential to speed up the treatment for cancer [3].

Gene editing techniques with CRISPR/Cas9 are based on creating double-strand breaks (DSBs) in specific genome sections, then repaired by cellular mechanisms. Depending on the cell state and the presence of a repair template, the cell machinery heals the DSB via one of two primary mechanisms: homology-directed repair (HDR) or non-homologous end joining (NHEJ) [2]. The specificity of CRISPR/Cas9 editing arises from the guide RNAs, which interact with target sequences via Watson-Crick base pairing [4]. Despite its benefits and potential, delivering CRISPR-Cas9 editing tools to targeted cells in vivo and avoiding or reducing unintended off-target effects remain key hurdles that are critical for therapeutic applications [5]. Hence, alternative gene-editing systems with improved accuracy like prime editors, cytosine base editors, and CRISPRon have started to emerge, and compared to conventional CRISPR/Cas9, they have improved efficiency and lower off-target effects than CRISPR/Cas9 itself [6, 7].

Moreover, precision or personalized medicine is based on treatments tailored to the patient’s cancer features. Obtaining cancer genomic profiles has become achievable due to the increased availability and affordability of NGS technologies that can uncover specific cancer traits [8]. Targeted therapy involves identifying mutations in signaling pathways and inhibiting existing or newly designed medications based on NGS profiles collected from various malignancies [9]. Hence results from NGS diagnosis could be used as a base by CRISPR/Cas tool for editing the mutated gene.

CRISPR/Cas9 genetic editing technology has been successfully employed for gene knock-in, gene knock-out, gene repair, and transcriptional regulation [10]. In this review, we have discussed the potential role of CRISPR/Cas9 and NGS in the advancement of personalized cancer medicine.

## Personalized medicine

Personalized or precision medicine aims in developing the treatment procedure tailored to the individual and eliminate the constraints of “one-size-fits-all” therapy [11–13]. In addition to broadening our understanding of cancer, NGS aided the development of personalized medicine, providing oncologists with a powerful tool to comprehend each patient’s disease and its unique genetic traits and whole-genome mutational status [12, 14]. NGS can detect tumor-specific mutations with the single-nucleotide resolution, allowing us to take advantage of this characteristic for target analysis [15].

The functional characterization of all annotated genetic elements in normal biological processes and disease has been a primary priority since the conclusion of the Human Genome Project [13]. The individual’s genetic profile can be analyzed using NGS and the mutations identified help us in identifying the therapeutic targets which can be edited with the help of CRISPR gene editing. The biomarkers from liquid biopsy make it easy to study the progression of the tumor.

**Fig. 1:**
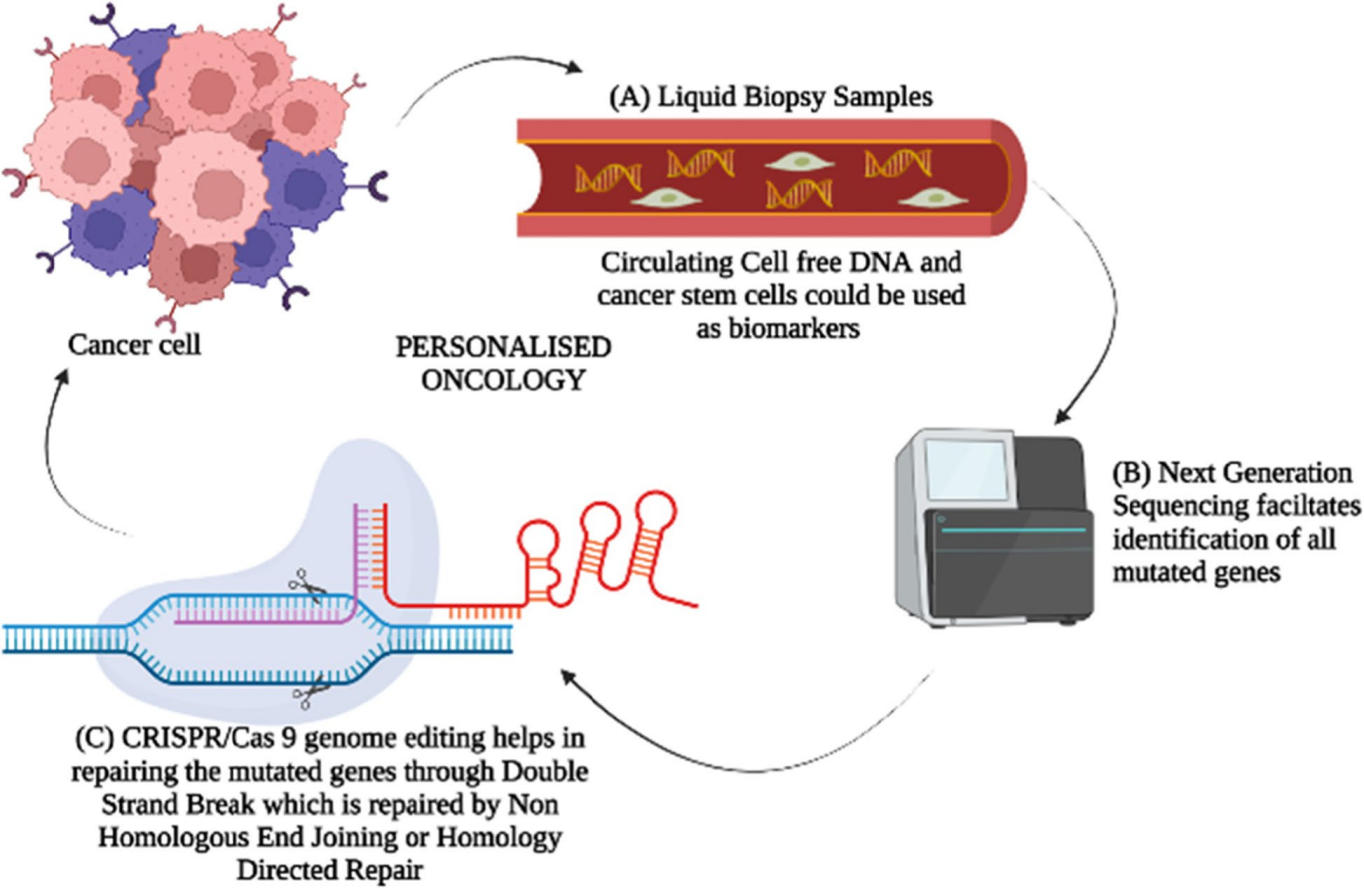
Represents the application of Next Generation Sequencing (NGS) and CRISPR/Cas9 in personalised medicine: **A** Cancer can be diagnosed without non-invasive biopsy samples with the help of circulation biomarkers (liquid biopsy) like cell free DNA and cancer stem cells. **B** The advancement of NGS has helped in identification of various mutation in the cancer cells that cannot be identified by other methods like PCR. This helps in personalising the treatment for cancer. **C** CRISPR/Cas9 genome editing tool locates the mutated gene and modifies it by creating double strand breaks which is corrected by non-homologous end joining or homology directed repair which is now being studied for personalised oncology

The main objective of personalized molecular medicine is to target very specific disease-causing genes while minimizing the danger of off-target consequences [10, 16]. CRISPR as a genome-editing technique could aid in the development of more efficient gene-targeted modification technology [17]. Figure 1 represents the application of Next Generation Sequencing (NGS) and CRISPR/Cas9 in personalized medicine.

To measure efficacy and determine treatment decisions, precision medicine approaches analyze patients’ circulating DNA (liquid biopsy), immunological markers, and other biologic aspects. Tumor and cell-free DNA profiling, immune markers, proteomic and RNA analyses can be used as diagnosis methods to determine the personalized treatment options [17]. Sequencing with NGS helps in genetic characterization, which can aid in target identification.

One of the important reasons for opting alternative therapeutic approaches and personalized medicine is drug resistance in cancer. Many tumors are initially responsive to chemotherapy, but they can develop resistance over time due to DNA mutations and metabolic changes that enhance drug inhibition and degradation. Reduced drug activation can potentially lead to cancer cells developing resistance to such treatments [18, 19]. In these cases, the mutation could be analyzed and treatment could be planned according to the genetic analysis in personalized medicine.

The efficacy of a pharmacotherapeutic drug is influenced by its molecular target and changes to that target, such as mutations or changes in expression levels. Signaling kinases like members of the epidermal growth factor receptor (EGFR) family are targeted by several anticancer drugs. In some malignancies, several of these kinases are constitutively active, which encourages uncontrolled cell proliferation. Over-activation of these kinases is usually caused by mutations; however, over-expression of these genes can sometimes have the same effect. HER2, a receptor tyrosine kinase in the EGFR family, is overexpressed in 30% of breast cancer patients, and treatment resistance can develop following long-term usage of inhibitors targeting this kinase. Factors like this make the need for an alternative therapeutic approach significant. Drug resistance can also be achieved via altering the signal transduction pathway that mediates drug activation, in addition to alterations in specific drug targets [19, 20]. Drug resistance is a serious impediment to advancement in the field of targeted therapy and it is worth noting that gene mutations causing medication resistance are now being identified by NGS analysis, which could lead to ways to restore sensitivity such as editing of drug-resistant genes using gene-editing tools.

### Liquid biopsy in diagnosis and monitoring of tumor

Liquid biopsies have ushered in a new era of precision medicine in the treatment of human cancer. Liquid biopsies may better reflect the genetic characteristics of all tumor subclones in a patient than tissue samples collected from only one tumor location [21]. Liquid biopsies have the potential to guide cancer treatment and provide the optimal strategy for personalizing treatment in precision medicine [22]. Furthermore, liquid biopsies can be taken at predetermined intervals to track therapy responses, medication resistance, cancer recurrence, and metastasis. The most successful biomarkers from such biopsies so far have been genomic biomarkers, although other biomarkers, such as protein assays and transcriptomics, are being developed and tested [23, 24].

Circulating cell-free tumor DNA (ctDNA) is one such promising biomarker from liquid biopsies that can support personalized treatment. Conventionally ctDNA can be assessed from various easily available physiological fluids, including blood, urine, and CSF. The two main methodologies for analyzing ctDNA are the detection of tumor-associated mutations or DNA methylation, and chromosome instability evaluation. NGS of gene panels, whole-genome sequencing (WGS), whole-exome sequencing (WES), RT-PCR, and other diagnostic platforms for ctDNA analysis are among the alternatives [25]. Treatment-selected mutations, such as *EGFR T790M* in non–small-cell lung cancer, *KRAS G12V* in colorectal cancer, and *BRAF V600E/ V600K* in melanoma have been detected using ctDNA, thus paving way for personalized treatment options [25–27]. Tumor NGS analyses genomes in the tissue biopsy, whereas ctDNA assesses DNA shed from numerous places, and ctDNA is associated with tumor load and can be detected at low levels [23].

ctDNA could be released into body fluids through passive mechanisms like tumor cell death and necrosis and active mechanisms like lymphocytes or complete tumors spontaneously releasing DNA, like the tumor DNA in exosomes. Many factors influence the amount of ctDNA in a patient’s bloodstream, including clinical stage, tumor size, cancer type, and cell turnover rate [28, 29]. Tumors without solid shape, necrosis, or enhanced mitotic activity may not be shedding ctDNA into the bloodstream or maybe shedding ctDNA at levels below current detection thresholds which is the main drawback in liquid biopsy samples [30].

The first blood-based colorectal cancer screening test authorized by the US Food and Drug Administration (FDA) is SEPT9 gene methylation detection. The European Medicines Agency and FDA have approved ctDNA-based epidermal growth factor receptor (EGFR) mutation testing for therapeutic recommendations in patients with non-small cell lung cancer (NSCLC) [31]. Thus, biomarkers from liquid biopsy aid in more frequent analysis, monitoring, early cancer screening, diagnosis, and prognosis because of their minimally invasive or non-invasive properties and high public acceptance.

### Next Generation Sequencing (NGS) in genetic profiling and target identification

The NGS technologies include whole-genome sequencing, whole-exome sequencing, RNA sequencing, reduced representation bisulfite sequencing, and chromatin immunoprecipitation sequencing [32]. For instance, breast cancer tumor sequencing can be used to determine which patients are most likely to benefit from aromatase inhibitor therapy. Serial genome sequencing can potentially reveal important details about disease activity and drug resistance [25].

Although tumor biomarkers have long been examined using Sanger sequencing or PCR, the introduction of NGS allowed for screening a larger number of genes in a single test. As a result, predictive biomarkers have emerged to help identify the appropriate patient populations for clinical studies. Furthermore, NGS allows researchers to discover the most frequent known variants and the long tail of unusual mutations that occur in fewer than 1% of patients and can provide useful information on medication sensitivity [12].

**Fig. 2:**
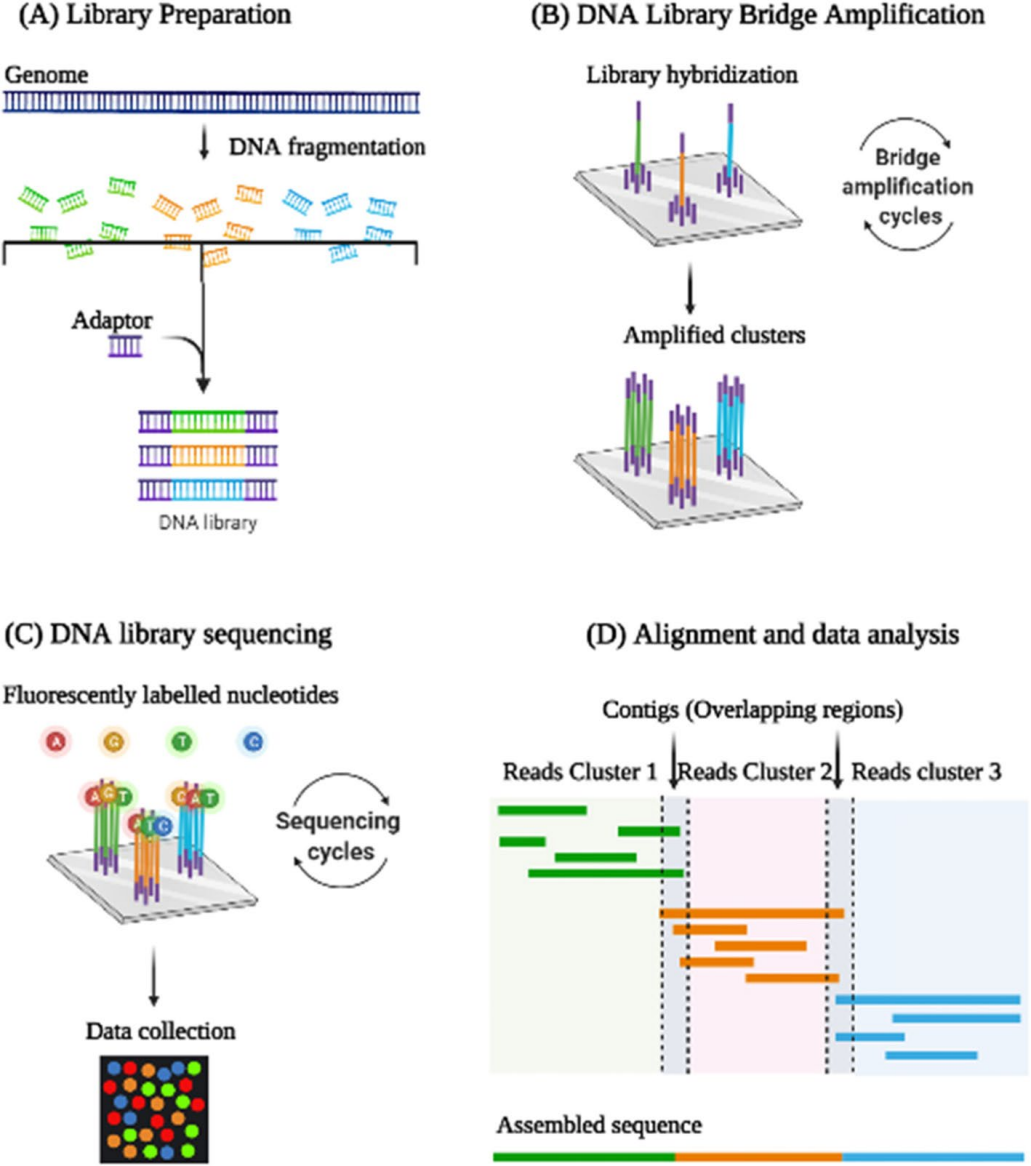
Represents the steps involved in Next Generation Sequencing: Library preparation and amplification, sequencing, and data analysis are the three important steps involved in NGS. The data analysis involves base calling, read alignment, variant identification, and variant annotation

Library preparation and amplification, sequencing, and data analysis are the three important steps involved in NGS. Illumina, Ion Torrent, and 454 Life Science are used in the sequencing [32]. Illumina works by bridge amplification where single molecules of DNA are linked to a flow cell and subsequently amplified locally into a clonal cluster, similar to how a single bacterium grows into a colony on a medium plate. The complementary DNA is then built one nucleotide at a time by sequencing by synthesis, and its identity is determined by an optical readout of fluorescently labeled nucleotides. On the other hand, single DNA molecules are cloned on a bead within an emulsion using the Ion Torrent platform. After that, the beads are placed on a semiconductor chip that has a matrix of individual pH sensors. A localized pH change identifies the sequenced nucleotide as the DNA clones undergo synthesis sequencing. Nanopores have been extensively used in recent years where single-stranded DNA is guided through a grid of protein nanopores, which collects the DNA sequence via electrical current interruptions. Nanopore sequencing does not necessitate preceding PCR amplification, albeit this is frequently done because of the high sample input required (> 500 ng). However, compared to other NGS technologies, the nanopore method has more sequencing mistakes, lower throughput, and higher per-read costs, which may restrict its utility for specific applications [33]. Tagged-Amplicon deep sequencing (TAm-seq), Cancer Personalized Profiling by deep sequencing (CAPP-Seq), Safe-Sequencing System (Safe-SeqS) are some of the technologies used to apply NGS to target panels [31, 34]. Figure 2 represents the steps involved in Next-Generation Sequencing.

Whole cancer genome sequencing has become possible, allowing researchers to identify genetic and epigenetic abnormalities that may play a role in tumor etiology as well as therapy resistance mechanisms. Driver mutations in the somatic cancer genome confer a selective growth advantage to malignancies that carry them, either directly or indirectly. Other modifications in the somatic cancer genome that persist through tumor progression but do not contribute to its growth are known as passenger mutations. The primary application of NGS in modern oncology is the detection of driver changes that result in oncogene addiction, as well as distinguishing between driver and passenger mutations [32, 35].

#### Clinical applications of NGS

NGS has been used to sequence ctDNA in order to create a cancer molecular profile. NGS of biomarkers from liquid biopsy samples can be used at any stage of cancer diagnosis and therapy, providing for non-invasive, real-time disease monitoring [31]. Both PCR and NGS can be used for diagnosis. Still, NGS is more accurate than PCR in identifying mutations in the genome and the former is capable of identifying mutations in addition to the mutations identified by the latter. A study by Tuononen et al [36] served as an example for the comparative studies between PCR and NGS, where NGS identified seven nonsynonymous single-nucleotide variations and one insertion-deletion variation that were not detectable by the real-time PCR methods. In addition, NGS can currently detect Minor Allele Frequency (MAF) of less than 1% which helps to differentiate the rare mutations in the genome. Molecular barcodes or unique molecular IDs can aid in improving sensitivity and reducing false negatives. With MAF under 0.1%, these approaches can detect 59% of stage I or II lung cancer patients. The complete analysis of the tumor is obtained from the NGS, which makes it easy to tailor the treatment options [32].

In thyroid cancer, NGS provides for a high-throughput sequencing study of several genomic changes simultaneously. Fine needle aspiration (FNA) cytology of the thyroid with ambiguous characteristics can be improved by NGS in thyroid cancer. It also aids patient care by allowing patients to be risk-stratified based on their cancer risk. In addition, NGS has been employed in papillary thyroid cancer molecular tumor classification and molecular prediction of recurrence and metastasis. NGS technology enabled the detection of additional somatic alterations in thyroid cancer, such as *MITF, JAK3, MDM2, IDH1*, *FLI1,* and others, in addition to the well-known *RAS*, *BRAF,* and *RET* mutations [37].

Moreover, NGS is increasingly being utilized to guide personalized treatment decisions and find new biomarker candidates for early lung cancer diagnosis. Whole Genome Sequencing, when performed on Stage I lung cancer, was useful in identifying recurrent somatic variation of *BCHE* and *TP53* in addition to the commonly identified *EGFR* mutation [38]. Moreover, when a patient with metastatic breast cancer did not react to numerous types of chemotherapy and only had a few months to live, NGS technology was used to detect somatic cell mutations, and immunotherapy was used to remove the tumor. Whole exome sequencing and RNA sequencing helped in the identification of 62 non-synonymous somatic mutations. Four of the mutant versions like *SLC3A2, KIAA0368, CADPS2,* and *CTSB* were targeted using tumor infiltrated lymphocytes [39]. Thus, this study served as an example that the genomic information of tumors detected by NGS can be used to identify patients who may respond to immunotherapy methods to induce the body’s immune system to attack and treat tumors [40].

Likewise, somatic mutations in the *TP53, PIK3CA*, and *GATA3* genes are common in breast cancer. However, the incidence of mutations in these genes varies among breast cancer subtypes. These variations in the mutation can be identified with the help of NGS, thus making it easier to classify the cancer subtype [41]. NGS has boosted the discovery of unknown genes that may be linked to improved treatment response and the development of drug resistance [40].

Interestingly, NGS can support the identification of new antigens for Chimeric Antigen Receptor (CAR) T cells therapy from WGS, WES, and RNA-seq data from T cells and tumor cells. As a result, NGS technology has become the foundation for developing targeted immunotherapy for cancer, as well as the development of individualized treatment plans for cancer patients [42]. Moreover, NGS can aid in the better understanding of tumors, tumor microenvironment, and T cells thus giving a clear idea of the treatment plan. Thus, the genetic analysis from NGS helps in identifying the target mutation which is responsible for disease progression. The data from NGS helps in further treatment planning for the patient and also helps in identifying the target gene for CRISPR.

#### Limitations of NGS

NGS is a powerful tool in analyzing genetic changes that correlate to clinical pathologies, yet there are certain limitations like analytic sensitivity of mutation detection where it is difficult to identify a low tumor percentage and lower mutation due to the heterogeneity of the tumor. Systemic errors and sequencing errors are common in NGS systems like Illumina. Current NGS platforms are not reliable in identifying the homologous genes, GC-rich region, and repetitive region. Interpretation of data from NGS is another major issue and the databases may not be accurate at all times. Copy number variations and structural variations require separate bioinformatics programs and hence many techniques are to be combined to read the NGS analysis [43, 44].

### Genome editing

Genome editing is a technique for altering genome sequences at specific sites to cause genetic alterations in organisms’ genome sequences. In recent years, gene editing has advanced significantly, and it is now widely used in targeted genome editing, with technologies like clustered regularly interspaced short palindromic repeats (CRISPRs) and CRISPR-associated nuclease 9 (Cas9), also known as the CRISPR/Cas9 system (2013) [16]. Before CRISPR/Cas 9, many other technologies such as Meganuclease (1994), zinc finger nucleases (ZFNs) (2003), transcription activator-like effector nucleases (TALENs) (2011) were used in gene editing [17].

#### CRISPR/Cas9

CRISPR/Cas9 can be used to modify genomes and investigate tumor occurrence, development, and metastatic pathways. Certain recent studies have used CRISPR/Cas9 to investigate tumor etiologies and therapies in novel ways [45]. For example, a study by Jandova et al [46] used CRISPR/Cas 9 based GLO-1 deletion in malignant melanoma cells and prostate carcinoma cells to study the disease etiology. Similarly, a study by Wang et al [47] used CRISP/Cas 9 for Hur knock out in melanoma cells for the parallel control of multiple tumor growth pathways.

The main prerequisite for Cas9 target selection is that the sequence should be proximal to the protospacer adjacent motif (PAM), which is situated downstream of the target gene [16]. Cas9 proteins are DNA-targeting endonucleases that are guided by RNA, and the two Cas9 nuclease domains, RuvC and HNH to the target site [17]. Through the complementary base pairing of gRNA and target genes, CRISPR/Cas9 initiates Cas9 cleavage before PAM, culminating in double-strand breaks (DSBs). As cell genomes can self-repair, CRISPR/Cas9-induced DSBs are repaired preferentially via error-prone non-homologous end joining, resulting in INDEL mutations in the target genes. INDEL mutations can cause frameshift mutations in the coding region, enabling gene transcription and translation to be disrupted and eventually leading to the knockout of certain target genes [48, 49].

The type II CRISPR/Cas system, which consists of three components: an endonuclease (Cas9), a CRISPR RNA (crRNA), and a transactivating crRNA (tracrRNA), is the most widely used method for gene editing. The guide RNA (gRNA) is a duplex structure formed by the crRNA and tracrRNA molecules that can be replaced by a synthetic fused chimeric single gRNA (sgRNA) to make CRISPR/Cas9 easier to use in genome engineering. The sgRNA comprises a unique 20-base-pair (bp) sequence that is meant to complement the target DNA site, and this must be followed by a short DNA sequence known as PAM, which is required for Cas9 protein compatibility [2, 50].

**Fig. 3:**
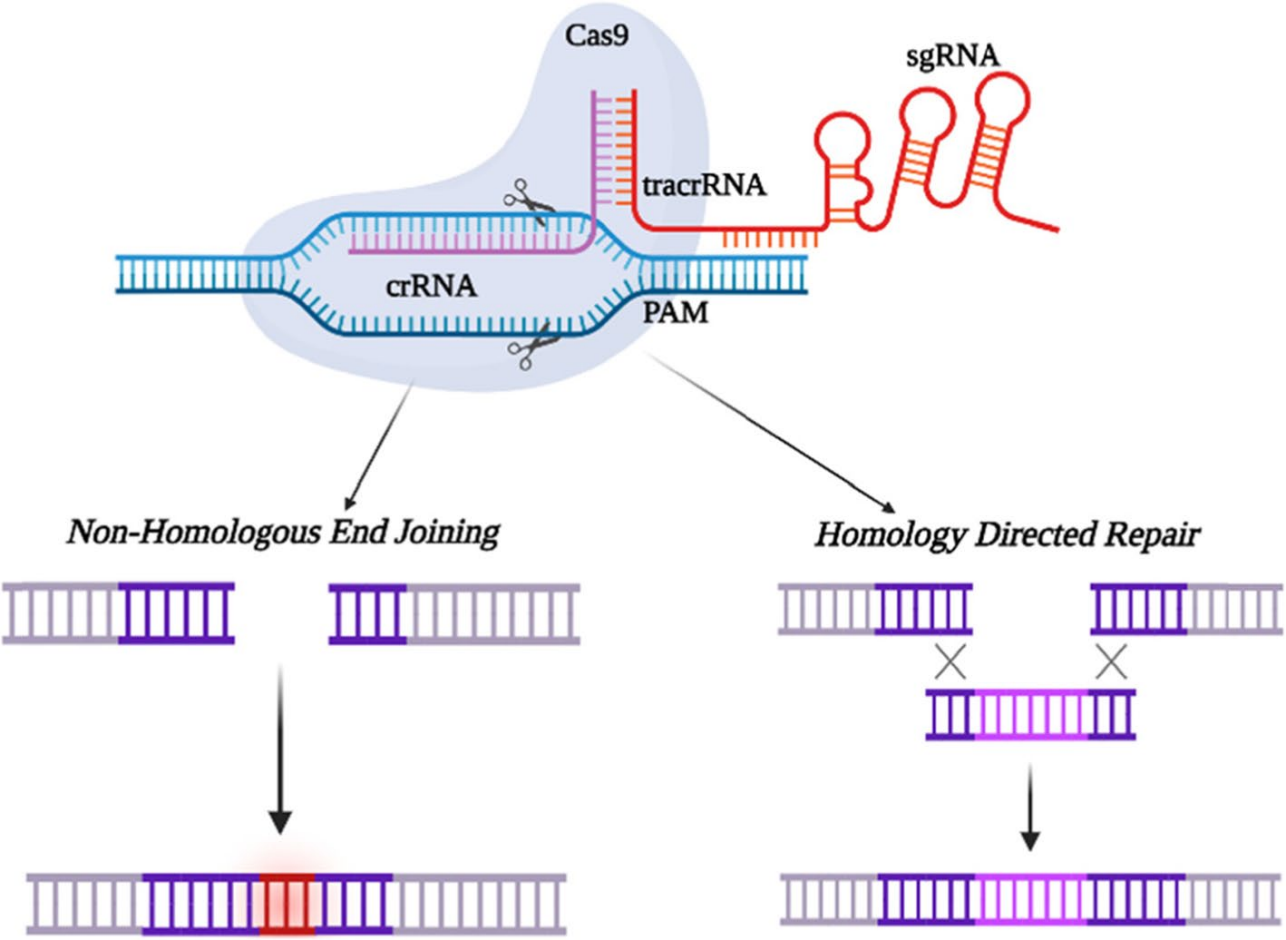
Represents the mechanism of CRISPR/Cas9: The CRISPR/Cas9 system consists of three components: an endonuclease (Cas9), a CRISPR RNA (crRNA), and a transactivating crRNA (tracrRNA). The guide RNA (gRNA) is a duplex structure formed by the crRNA and tracrRNA molecules. The sgRNA comprises a unique 20-base-pair (bp) sequence that is meant to complement the target DNA site, and this must be followed by a short DNA sequence known as PAM, which is required for Cas9 protein compatibility. Cas9 nuclease is guided by sgRNA which causes Double Strand Breaks (DSB) around the PAM. Non-Homologous End Joining (NHEJ) or Homology Directed Repair (HDR) are the two routes through which the DNA repair machinery is activated to repair DSBs

NHEJ or HDR are the two routes through which the DNA repair machinery is activated to repair DSBs. NHEJ, as a major and effective repair process, connects the two ends of DSBs to repair lesions and frequently causes tiny insertions or deletions (indels) in gene knockout tests. HDR, on the other hand, is a slow but accurate repair method that requires the presence of a DNA template. Based on the DNA template and the position of homology arms, this repair mechanism can be employed for precise genome remodeling at the site of DSBs for gene knock-in [51]. The activity of the DSB repair pathways is important in modulating CRISPR-Cas9 editing rates. Figure 3 represents the mechanism of CRISPR/Cas9. The expression of Cas9 protein in targeted cells can be induced by DNA or mRNA delivery, resulting in Cas9-mediated gene editing. Till date, viral vectors like adeno-associated virus (AAV) have been the most extensively used vectors in diverse investigations for efficient in vivo delivery of CRISPR-Cas9 [5].

#### Therapeutic applications of CRISPR/Cas9 in cancer

The genetic profile of cancer and immune cells from NGS can help researchers better understand their molecular heterogeneity and interactions in the tumor microenvironment, which can help improve CRISPR/Cas9 mediated immunotherapy efficacy. Since CRISPR/Cas9 may be used to silence or damage any desired genetic location, combining this technology with immunotherapy like Chimeric Antigen Receptor (CAR) T cell therapy could be a strategy for cancer treatment success [52, 53]. One of the most eye-catching applications of CRISPR-Cas9 technology in cancer immunotherapy is universal CAR-T [54]. Due to their potential to specifically target and trigger an immune response in cancer, three types of immunotherapy methods, CAR T cell treatment, checkpoint inhibitors, and vaccine immunotherapy, have emerged as forerunners of cancer immunotherapy [35]. The generation of autologous CAR T-cells has some drawbacks, which included time consumption, cost, as well as difficulty in collecting high quality and quantity of T cells from patients with critical diseases, which limits the use of these modified T cells to a small number of patients. These limitations have prompted the development of universal T cells, which are CRISPR modified allogeneic T cells derived from healthy donors and have the potential to overcome these limitations and make them available to a large number of patients [52].

In clinical trials, cancer immunotherapy, such as the use of immune checkpoint inhibitors and CAR T-cell therapy, has shown strong anticancer effectiveness [55]. CRISPR-Cas9-mediated *PD-1, PDL-1*, or *CTLA-4* gene deletion is an effective strategy for breaking T-cell-based adoptive therapy tolerance in tumor therapy. The first case of Cas9 use in a clinical trial was reported in 2016, where Cas9-engineered PD-1-deleted T cells were injected into a patient with aggressive non-small cell lung cancer. Safety, feasibility, and efficacy were tested over the patient population and the outcomes were satisfactory with an overall survival median of 42.6 weeks and 0.05% off-target events. All patients had tumor progressed by the end of January 2020. 11 (91.7%) of the 12 patients died as a result of tumor development. The one patient who remained was still undergoing treatment. The research was concluded with no death due to the result of the treatment [56].

**Fig. 4:**
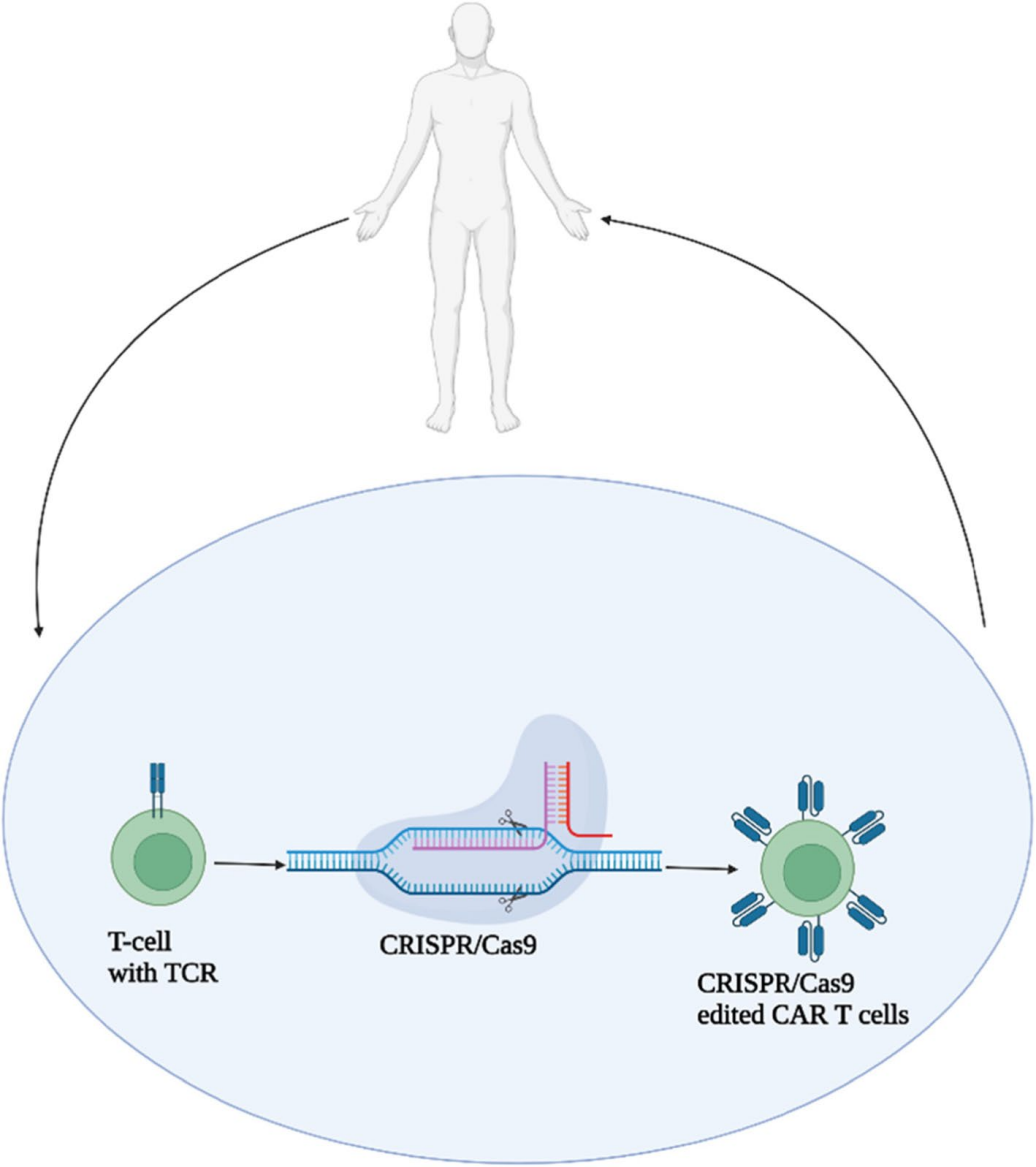
Represents the mechanism of CRISPR/Cas9 mediated CAR T cell immunotherapy: The T cells from the patients are removed and genetically modified by CRISPR/Cas9 mediated knock-in/knock-out mechanism, giving rise to the Chimeric Antigen Receptor T cells that contain an intracellular chimeric signalling domain that can activate T cells and an external single-chain variable segment that can recognise tumour antigens precisely. These CRISPR/Cas9 edited CAR T cells are again introduced into the host as treatment

CARs have an intracellular chimeric signaling domain that can activate T cells and an external single-chain variable segment that can recognize tumor antigens precisely. Patients with various hematological malignancies, such as leukemia and lymphomas, have had favorable therapeutic outcomes with these genetically engineered T cells containing tumor-targeting receptors [57]. However, CARs can cause cytokine release due to endothelial dysfunction and it varies from moderate to severe in various patients. There are also chances of neurotoxicity which is due to the cytokine storm [58]. Hence T cell receptors (TCRs) have also been evaluated. For example, a phase I human trial (ClinicalTrials.gov NCT03399448) was established to see the safety and feasibility after infusing autologous NY-ESO-1 TCR modified T cells which are edited with CRISPR-Cas9 to knock out endogenous TCRα, TCRβ (for specificity), and PD-1 (anti-tumor activity) into the patients. The cancer cells were extracted, modified, and then put back into the patient. TCRs being less likely to cause cytokine release syndrome than CARs, they were utilized instead of CARs. This study recruited only 3 patients but provides feasibility and the study revealed that the edited T cells retained up to 9 months proving that the multiplex CRISPR-Cas9 genome editing is feasible for therapeutic scale purposes [59]. Figure 4 represents the mechanism of CRISPR/Cas9 mediated CAR T cell immunotherapy.

The application of CAR-T cell immunotherapy and CRISPR/Cas9 has been studied in multiple myeloma, glioblastoma, leukemia, and so on with promising results in animal models. Yet, reaching target infusion doses during cell harvest due to a decrease in lymphocyte viability, following electroporation and genome alteration, which promotes genetic instability, is a potential limitation in the manufacture of CRISPR/Cas9-edited CAR T cells. The potential for Cas9-sgRNA binding and cleavage of sequences that are highly similar to the target DNA sequence is another hindrance to clinical translation of CRISPR/Cas9-edited CAR T cells. This could result in mutations at undesirable locations in the DNA [60]. Thus CRISPR/Cas9 is in constant research to overcome the limitations and provide its potential therapeutic properties.

##### CRISPR/Cas 9 in oncolytic virus production

CRISPR/Cas 9 is used in the production of oncolytic viruses which lack virulence but are still capable of lysing cancer cells [61]. The production of herpes simplex virus type 1 variants with significant lytic capabilities, engineered by deletion of the ICP34.5 neurovirulence and ICP6 (UL39) (ribonucleotide reductase) genes, is one example of genomic alteration used for immunotherapy applications. Another example is the deletion of ICP6 to offer replicative selectivity for cells with inactivation of the p16INK4A tumor suppressor gene, which is one of the most common defects in cancer [62]. The wild-type version of the DNA tumor virus adenovirus encodes a protein (E1A) that binds pRb, releasing the transcription factor E2F and thereby halting the cell cycle. The release of E2F also causes a coordinated activation of viral genes, which results in the formation of new virions, the lysis of infected cells, and the dissemination of the new virus. The E1A gene has been removed from oncolytic adenoviruses to limit replication and promote safety in wild-type cells, as cancer cells generally have genetic abnormalities in the Rb pathway [2, 63].

##### CRISPR with deactivated cas9

Deactivated Cas9 (dCas9), which is catalytically inert, can be recruited to specific target DNA locations by gRNAs and utilized to activate or repress specific target genes when linked to transcriptional activation or inhibitory domains [64]. The attachment of dCas9 to histone modifiers and proteins involved in DNA methylation to execute targeted “epigenome editing” offers enormous potential in cancer therapeutic applications. Because many epigenetic variables are implicated in a variety of cancers, including acute lymphoblastic leukemia (ALL) and Ewing sarcoma, targeting the epigenetic regulatory machinery could be a useful way to improve cancer treatment outcomes [2]. In a study by Batsche E et al [65], dCas9 tool was used to study the role of methylation DNA in the alternative splicing of HCT116 colon cancer cells. They also examined the influence of DNA methylation in MCF10A breast cancer model and ALL patients. dCas9 tool was used to target epigenetic regulation, which adds data to their potential use in cancer therapeutics.

Another study by Abraham KJ et al [66] has used dCas9 to disrupt the epigenetic regulation in Ewing sarcoma cell lines, where dCas9 was used as a knock-out tool to establish the role of RNA polymerase II in ribosome biogenesis. CRISPR activators (CRISPRa) and inhibitors (CRISPRi) are created by fusing dCas9 with different transcription regulatory domains to activate or suppress the expression of a target gene [67].

##### CRISPR prime editors

Prime editing (PE) is an adaptable and specific genome editing method that uses a catalytically impaired Cas9 endonuclease with a genetically engineered reverse transcriptase which is programmed with a prime editing guide RNA (pegRNA) that specifies the target site and encodes the desired edit to directly write new genetic information into a specified DNA site. One of the studies used prime editing in order to lower off-target effects with good efficiency [6].

Based on the template sequence encoded within the pegRNA, nucleotide substitutions, local insertions, and deletions within the genome are made possible by PE. Another investigation used an NLS-optimized SpCas9-based PE to boost genome editing efficiency in fluorescent reporter cells and cultivated cell lines at endogenous loci. The researchers used AAVs to deliver a split-intein prime editor to the mouse liver, demonstrating that this method can fix a pathogenic mutation [68]. PE tends to reduce off-target effects due to their short duration of activity. There are not enough studies to prove that PE is efficient and lowers the off-target effects since most of the studies are done in vitro rather than in vivo. Only limited studies have focused on prime editing and in a study by Petri K et al [69], prime editing experimented in the zebrafish embryo exhibited 30% frequency and many unintended insertions, deletions, and incorporation of pegRNA scaffold. Thus, PE is in the early stages of research and has certain limitations towards its use in cancer therapeutics. We nonetheless anticipate prime editing on cancer cells will be a fertile area of investigation over the next few years.

##### CRISPR Base editor

Adenine Base editors (ABE) and Cytosine Base editors (CBE) are both studied for their potential role in disease modeling and therapeutics. A Cas enzyme for programmed DNA binding and a single-stranded DNA modifying enzyme for targeted nucleotide change make up base-editors. Uracil is formed when cytosine is deaminated and it base pairs with thymidine in DNA. The fusion of uracil DNA glycosylase inhibitor (UGI) and uracil N-glycosylate (UNG) suppresses the activity of uracil N-glycosylate (UNG), improving the cytosine base-editing efficiency in human cells. Inosine is formed when adenosine is deaminated, and it has the same base-pairing preferences as guanosine in DNA. All four transition mutations can be installed using cytosine and adenine base editing together [70–72].

A study was performed based on CBEs which is also a genome editing tool that contains a cytidine deaminase attached to the catalytically inactive Cas9. Both human cells and *Escherichia coli* were tested with CBE and analyzed with NGS to identify off-target effects. The results suggested that CBEs exhibit lower off-target effects and efficient on-target editing [7].

One of the studies used iPSCs to evaluate BEs, and the results show that employing BEs rather than nuclease-based HDR can improve the correction of disease-causing mutations. The increased editing frequency should make it easier to find clones that have the necessary change. Off-target editing of DNA and RNA is becoming an issue with Bes also and these side effects raise the danger of undesired modifications, and iPSCs may be especially sensitive, as expression changes caused by RNA cross-editing could result in the reduction of pluripotency and differentiation. Base editing is currently limited to single base alterations and only a few nucleotide changes are possible [73]. Both BE and PE are only for short-term usage and cannot guarantee a lower off-target effect, hence further studies could lead to their potential role in therapeutics.

##### CRISPRon

In a study by Xiang X et al [74], it was proved that CRISPRon improves CRISPR applications by predicting gRNA efficiency more accurately than existing methods. SgRNA and dCas9 protein were coupled with a transcriptional activation domain in the CRISPR-ON system. CRISPR-ON has the properties of stability and accuracy, and it can be used to screen for gain-of-function (GOF) at the genome-scale [75]. Researchers used the CRISPR-ON method to upregulate KLF4 expression in a study, which looked at the effect and mechanism of KLF4 in the carcinogenesis and development of urothelial bladder cancer (UBC). The researchers concluded that KLF4 overexpression driven by CRISPR-ON reduces carcinogenesis and that the CRISPR-ON technology could 1 day be used to treat UBC [76]. Since there are limited studies on the efficiency of CRISPRon, it is unknown whether this approach can be employed widely or if it will be limited in its application based on cell types and methylation promoter statuses.

#### Limitations of CRISPR/Cas9

There are still several factors to be addressed for the complete clinical application of CRISPR/Cas9 in terms of efficacy and safety, such as the fitness of altered cells, editing efficiency, delivery methods, and potential off-target effects. Edited cells frequently have fitness defects, such as a reduced ability to proliferate and differentiate, resulting in inadequate therapeutic effects [77]. Cancer cells, on the other hand, have an edge in terms of growth, including rapid proliferation and prolonged survival. This will necessitate great editing efficiency for CRISPR-Cas9. Since CRISPR/Cas 9 can induce p53 mutation there are chances that in altered cells, p53 will spontaneously mutate, and Cas9 can trigger a p53-mediated DNA damage response [5, 78]. Further studies on methods to reduce the off-target effects could finally bring a clinical breakthrough for CRISPR/Cas9 in the treatment of cancer and many other diseases [79].

Continued genomic modification increases the likelihood of off-target cleavage and reduces editing selectivity, potentially leading to undesired mutations and toxicity. Endonuclease-induced off-target events should be reduced when using CRISPR-Cas9 in vivo since indel creation at undesired loci can impact cell viability or promote cancer [5]. The off-target cleavage sites from the gRNA/SpCas9 endonuclease ribonucleoprotein (RNP) system were first found in vitro on genomic DNA isolated from an animal model. The verification of in vivo off-targets approach has shown that the developed gRNAs are highly unlikely to cause off-target effects. But it is better to go for bioinformatics before in vivo and in vitro since bioinformatic pipelines and web-based algorithms are available for CRISPR/Cas systems to provide recommendations for optimal guide RNA (gRNA) design, reducing predictable off-target actions [14, 16].

The first-ever CRISPR-Cas9 project on humans was performed by a Chinese researcher He Jiankui in 2018, which turned out to be an ethical crime. He developed twin girls with CCR5 gene edition to become resistant to HIV, cholera, and smallpox. But, having a major limitation of off-target binding, CRISPR-Cas9 led to the mosaic genes [80].

## Future perspectives

Precision oncology is a branch of oncology that focuses on gene-directed, histology-agnostic treatments that are tailored to each patient based on biomarker analyses. Tumor and cell-free DNA profiling using NGS, as well as proteome and RNA analyses and a better understanding of immunological systems, are all helping to improve cancer treatment choices [81]. The intricacy of tumor biology is a key barrier in the therapeutic management of patients with advanced metastatic disease. The biggest challenge in genome editing is to reduce the potential off-target effects with increased CRISPR-Cas9 specificity.

Our understanding of the immune cell and tumor interaction is likely to increase and be translated for the development of personalized treatment as the cost and accuracy of NGS applications such as WGS, WES, RNA-seq, and ChIP-seq at the bulk tissue and single-cell level decreases. New NGS technologies are also rapidly advancing, with NICHE-seq generating a lot of buzz among cancer researchers because of its potential to add spatial information to single-cell RNA-seq data from tumors modified to express photoactivable GFP protein. Novel techniques, such as RNA-seq, offer the potential to uncover genetic causes of cancer that might go undetected by genomic DNA screening. The methods employed to track off-target activities are identified using a variety of ‘OMIC’ technologies, such as ChIP-sequencing (ChIP-seq), that detects the modified proteins’ direct genome-wide binding events [16]. Recent improvements in single-cell RNA-seq and ChIP-seq techniques promise to reveal transcriptome and epigenetic heterogeneity in cancer cells and immune cells at the single-cell level. Further research on these techniques could bring a new light in sequencing studies.

In recent years, neoantigens are studied for their potential role in cancer immunotherapy. Pipelines for neoantigen prediction are rapidly being built and optimized, and developments in NGS techniques and applications promise to address many of the existing issues in neoantigen prediction and speed up the development of effective cancer vaccines [11]. The current research has summarized the uses of CRISPS/Cas9 and NGS in the personalized treatment of cancer. The possible outcomes of personalized medicine outweigh the disadvantages, hence, further studies of genome editing, sequencing, liquid biopsy biomarkers in cancer treatment are warranted.

## Conclusion

Cancer personalized medicine is still being developed by researchers and CRISPR-Cas9 is still in the earlier stage of research. All the challenges in genome editing are to be solved before implementing in humans. But as outlined, CRISPR/Cas9 has been applied to human carcinoma cells like melanoma and prostate cancer and also tested in non-small cell lung carcinoma, leukemia, and lymphoma. Hence CRISPR/Cas9 can be used to alter the genes that are identified by NGS which can identify mutations that are not identified by other means and makes it easier to understand the molecular mechanism behind the tumor. Yet, the cost has been a problem in NGS which has to be made economically available for everyone. Genome editing in personalized cancer treatment is likely to revolutionize cancer therapy in the twenty-first century, and it will be important to ensure that technical and economic barriers to access these life-changing technologies are as low as possible.

## Data Availability

Not applicable.

## Abbreviations

AAV: Adeno-Associated Virus
CAPP-Seq: Cancer Personalized Profiling by deep sequencing
CAR: Chimeric Antigen Receptor
Cas9: CRISPR associated nuclease 9
CBE: Cytosine Base Editor
CRISPR: Clustered Regularly Interspaced Short Palindromic Repeats
crRNA: CRISPR RNA
ctDNA: Cell-free tumor DNA
dCas: Deactivated Cas 9
DSB: Double Strand Breaks
FDA: Food and Drug Administration
FNA: Fine Needle Aspiration
gRNA: Guide RNA
HDR: Homology Directed Repair
MAF: Minor Allele Frequency
NGS: Next Generation Sequencing
NHEJ: Non-Homologous End Joining
NSCLC: Non-Small Cell Lung Carcinoma
PAM: Protospacer Adjacent Motif
PCR: Polymerase Chain Reaction
PE: Prime Editor
pegRNA: Prime editor guide RNA
RNP: Ribonucleoprotein
sgRNA: Single guide RNA
TALEN: Transcription Activator-Like Effector Nucleases
TAm-seq: Tagged-Amplicon deep sequencing
TCR: T-Cell Receptor
tracrRNA: Transactivating crRNA
WES: Whole Exome Sequencing
WGS: Whole Genome Sequencing
ZFN: Zinc Finger Nucleases
